# Smad2Δexon3 and Smad3 have distinct properties in signal transmission leading to TGF-β–induced cell motility

**DOI:** 10.1016/j.jbc.2022.102820

**Published:** 2022-12-20

**Authors:** Takashi Yokoyama, Takahito Kuga, Yuka Itoh, Shigeo Otake, Chiho Omata, Masao Saitoh, Keiji Miyazawa

**Affiliations:** 1 Department of Biochemistry, Graduate School of Medicine, University of Yamanashi, Yamanashi, Japan; 2 Research Training Program for Undergraduates, Graduate School of Medicine, University of Yamanashi, Yamanashi, Japan; 3 Center for Medical Education and Science, Graduate School of Medicine, University of Yamanashi, Yamanashi, Japan

**Keywords:** ARHGAP24, cell motility, EMT, Smad, TGF-β, cDNA, complementary DNA, EMT, epithelial–mesenchymal transition, GAP, GTPase-activating protein, TGF-β, transforming growth factor-β

## Abstract

In mammalian cells, Smad2 and Smad3, two receptor-regulated Smad proteins, play crucial roles in the signal transmission of transforming growth factor-β (TGF-β) and are involved in various cell regulatory processes, including epithelial–mesenchymal transition–associated cell responses, that is, cell morphological changes, E-cadherin downregulation, stress fiber formation, and cell motility enhancement. Smad2 contains an additional exon encoding 30 amino acid residues compared with Smad3, leading to distinct Smad2 and Smad3 functional properties. Intriguingly, Smad2 also has an alternatively spliced isoform termed Smad2Δexon3 (also known as Smad2β) lacking the additional exon and behaving similarly to Smad3. However, Smad2Δexon3 and Smad3 signaling properties have not yet been compared in detail. In this study, we reveal that Smad2Δexon3 rescues multiple TGF-β–induced *in vitro* cellular responses that would become defective upon *SMAD3* KO but does not rescue cell motility enhancement. Using Smad2Δexon3/Smad3 chimeric proteins, we identified that residues Arg-104 and Asn-210 in Smad3, which are not conserved in Smad2Δexon3, are key for TGF-β–enhanced cell motility. Moreover, we discovered that Smad2Δexon3 fails to rescue the enhanced cell motility as it does not mediate TGF-β signals to downregulate transcription of *ARHGAP24*, a GTPase-activating protein that targets Rac1. This study reports for the first time distinct signaling properties of Smad2Δexon3 and Smad3.

Transforming growth factor-β (TGF-β) is a pleiotropic cytokine involved in the regulation of various cellular processes during embryogenesis as well as adult tissue homeostasis. TGF-β–derived signaling is transmitted through both the Smad and non-Smad signaling pathways. In the Smad signaling pathway, ligand stimulation induces the TGF-β type I receptor–mediated phosphorylation of Smad2 and Smad3, receptor-regulated Smads (R-Smads), at their C termini, leading to their heterotrimeric complex formation with Smad4, and translocation into the nucleus. The Smad complex subsequently upregulates or downregulates gene expression, through binding to genomic regulatory regions, in cooperation with Smad-binding transcription factors and coactivators/corepressors (1). Smad-binding transcription factors, collectively “Smad cofactors,” are thought to assist the selective and stable binding of Smad proteins to the genomic DNA, thereby contributing to context-dependent gene expression in the target cells (2). Alternatively, R-Smads can affect gene expression *via* the repression or derepression of other transcription factors *via* physical interaction (3, 4), where the DNA-binding activity of Smads could be dispensable.

Five mammalian R-Smad proteins have been identified so far, among which Smad2 and Smad3 transmit TGF-β-, activin-, and Nodal-related signaling, whereas Smad1, Smad5, and Smad8 transmit signaling from bone morphogenetic proteins (5). Although Smad2 and Smad3 share 92% sequence identity, they are not functionally redundant: they exhibit distinct binding partners and oligomeric states, nuclear import mechanisms, DNA-binding properties, as well as different target genes and spatiotemporal expression patterns (6, 7). The pathophysiological importance of Smad2 and Smad3 is context dependent: in cells cultured *in vitro*, Smad3 appears to be crucial in several TGF-β–induced cell responses. In contrast, Smad2 is indispensable for *in vivo* embryonic development as its KO induces embryonic lethality in mice, whereas Smad3 KO mice are viable (6). Smad2-mediated transcriptional regulation has thus been well explored in the embryonic developmental context. In FoxH1-related developmental signaling, Smad2 is essential for mesendoderm gene induction, whereas Smad3 plays only a limited role in the process (8, 9). In immune cell development, Smad2 regulates positively, whereas Smad3 regulates negatively Th17 differentiation (10, 11, 12). In skin squamous cell carcinoma and non–small cell lung cancer cells, Smad2 suppresses, whereas Smad3 promotes cancer formation, malignant progression, and metastasis (13, 14, 15, 16). In addition, Smad2 mutations are found in various cancers at a low frequency, whereas Smad3 mutations are rare and could be found only in colon cancers (17). Although Smad2 and Smad3 are independently recruited to often distinct genomic regions upon TGF-β stimulation in A549 lung cancer cells (16), the underlying mechanisms of the distinct Smad2 and Smad3 functions remain mostly unclear.

The most striking biochemical difference between Smad2 and Smad3 is the interaction of Smad3, but not full-length Smad2, with CAGA motif–containing DNA sequences upon C-terminal phosphorylation. Exon 3 of Smad2 is key to this process, encoding 30 amino acid residues located at the N-terminal side of the DNA-binding β-hairpin structure to interfere with Smad2 DNA binding (18, 19). Moreover, exon 3 affects the cytoplasmic localization of Smad2 (20). Consistently, Smad2Δexon3 (Smad2β), an alternatively spliced Smad2 isoform (21), exhibits DNA-binding ability and behaves similarly to Smad3 in biochemical assays including CAGA-Luc reporter and electrophoretic motility shift assays (18, 19). In *Xenopus* animal cap assay, where Smad2 and Smad3 exert distinct target gene induction, *Xenopus* Smad2Δexon3 activity was more similar to that of *Xenopus* Smad3 than that of *Xenopus* Smad2; *Xenopus* Smad2Δexon3 thus partially loses unique *Xenopus* Smad2 features (22). Smad2Δexon3 and Smad3 are reportedly functionally interexchangeable during early embryonic development in mice (23), although Smad2Δexon3 is significantly less efficient compared with full-length Smad2 in the nodal stimulation–related mesendoderm gene induction of embryoid body cells (9).

In the present study, we examined if Smad2Δexon3 and Smad3 are functionally distinct during epithelial–mesenchymal transition (EMT), a process involved in tumor progression (24). Using the CRISPR–Cas9 technology, we prepared *SMAD2 exon 3/SMAD3*-double KO A549 cells that exclusively express the Smad2Δexon3 isoform as R-Smad that transmits signals from TGF-β/activin/Nodal. Smad2Δexon3 could mediate multiple TGF-β–induced and EMT-associated cellular responses, including cell morphological changes, E-cadherin downregulation, stress fiber formation, although it failed to mediate cell motility enhancement. The signaling difference was attributed to Arg-104 in the β4 region of the MH1 domain and Asn-210 in the linker region of Smad3. Both amino acid residues are required for downregulating *ARHGAP24*, encoding the GTPase-activating protein (GAP) Fil-GAP that targets Rac1. To the best of our knowledge, this study describes first the functional differences between Smad2Δexon3 and Smad3 either *in vitro* or *in vivo*.

## Results

### Establishment of SMAD2 exon 3/SMAD3-double KO A549 cells

**Figure 1:**
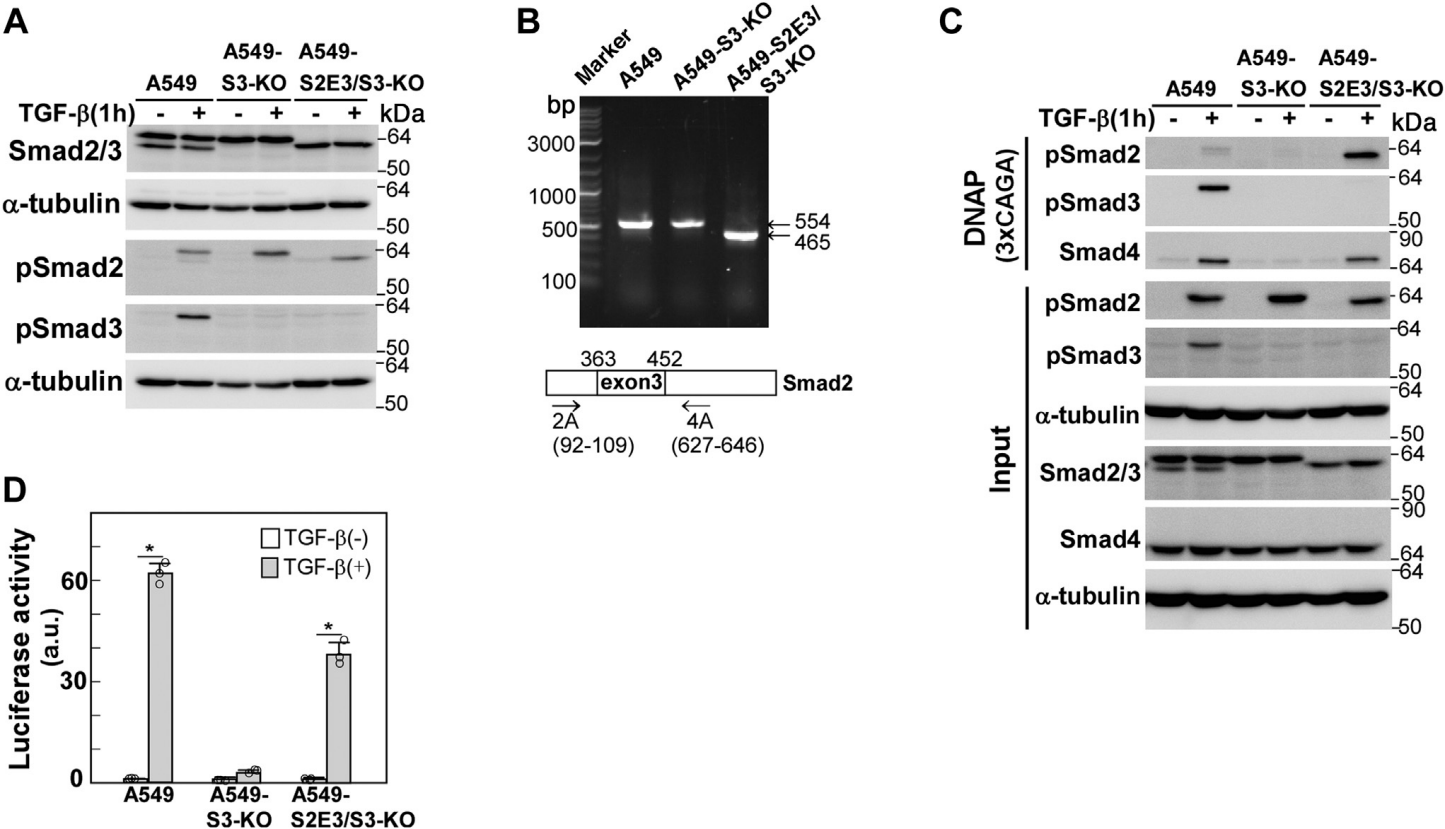
**The establishment of *SMAD2 exon3/SMAD3-*double KO A549 cells**. *SMAD2 exon 3/SMAD3-*double KO A549 cells (A549-S2E3/S3-KO) were prepared from *SMAD3* KO A549 cells (A549-S3-KO) using CRISPR–Cas9-mediated genome editing. *A*, the expression and TGF-β–induced phosphorylation of Smad2/3 were verified by immunoblotting (wet tank transfer) with the indicated antibodies; α-tubulin was used as a loading control. *B*, PCR analysis of *SMAD2* transcripts using primers (2A and 4A) corresponding to the sequences in exons 2 and 4. We confirmed that the 465-bp product was amplified from Smad2Δexon3 by DNA sequencing. *C*, DNA affinity precipitation (DNAP) assay was performed using biotinylated 3xCAGA as a probe. Total cell lysate (input) and proteins bound to the probe were analyzed by immunoblotting (wet tank transfer) with the indicated antibodies; α-tubulin was used as a loading control. *D,* a luciferase reporter assay in A549-S2E3/S3-KO cells, carried out using (CAGA)_12_-MLP-Luc as a reporter plasmid. TGF-β1 stimulation for 18 h. Error bars represent the SD (n = 3). The *p* values were determined by Student’s *t* test. ∗*p* < 0.01. One representative result from two independent experiments is shown (*C* and *D*). TGF-β, transforming growth factor-β.

We previously established *SMAD3* KO A549 (A549-S3-KO) cells using CRISPR–Cas9-mediated genome editing (4). In these A549-S3-KO cells, TGF-β failed to induce most EMT-associated cellular responses, including cell morphological changes, E-cadherin downregulation, actin stress fiber formation, and enhanced cell motility, although cells still expressed full-length Smad2 (25). In order to examine if Smad2Δexon3 could rescue the TGF-β–related cellular responses eliminated by the *SMAD3* KO, we further deleted exon 3 of *SMAD2* and established *SMAD2 exon3/SMAD3*-double KO A549 (A549-S2E3/S3-KO) cells. The *SMAD2* exon 3 deletion or insertion was introduced as shown in Fig. S1, resulting in premature termination codons. Therefore, mature transcripts including exon 3 yielded truncated proteins, whereas those excluding exon 3 successfully encoded the Smad2Δexon3 protein (9, 19). The cells no longer expressed the full-length Smad2. Instead, they exclusively expressed Smad2Δexon3, verified by immunoblotting (Fig. 1*A*) and RT–PCR analyses (Fig. 1*B*).

We performed a DNA affinity precipitation assay using the Smad-binding 3xCAGA probe. Our results revealed that the probe precipitated Smad3 and Smad4 upon TGF-β stimulation in parental A549 cells, whereas no Smad4 precipitation could be detected in *SMAD3*-KO cells, which could be recovered in A549-S2E3/S3-KO cells expressing Smad2Δexon3 (Fig. 1*C*). In addition, the TGF-β–responsive CAGA_12_-MLP-Luc reporter, which was almost inactive in A549-S3-KO cells, was activated in A549-S2E3/S3-KO cells (Fig. 1*D*). These biochemical properties were consistent with a previous report on overexpressed Smad2Δexon3 (19).

### Smad2Δexon3 rescued cellular responses to TGF-β attenuated in SMAD3-KO A549 cells except for enhanced cell motility

**Figure 2:**
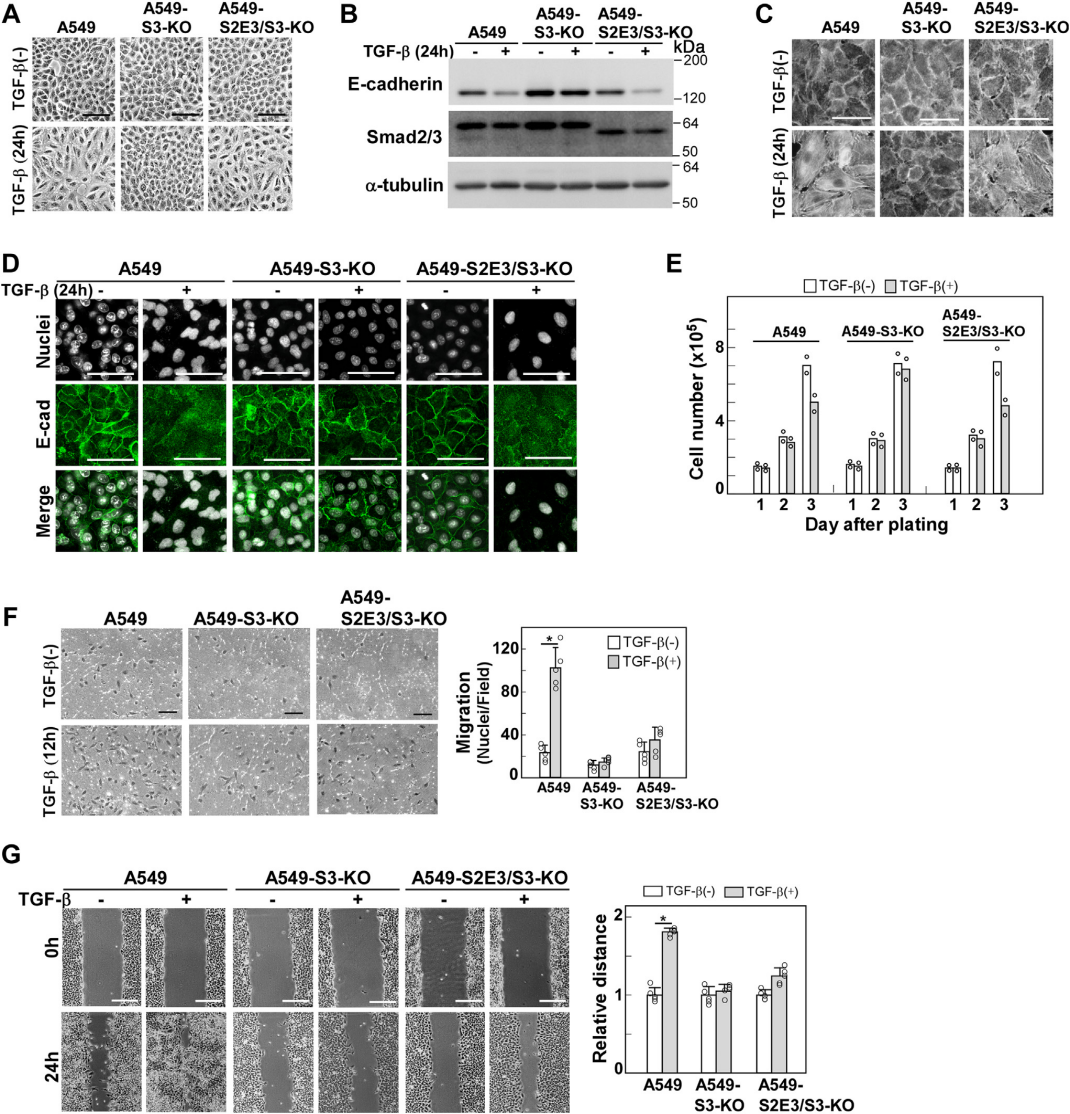
**Cellular responses of A549-S2E3/S3-KO cells exclusively expressing Smad2Δexon3 as an R-Smad.***A*–*G*, A549 cells, A549-S3-KO cells, or A549-S2E3/S3-KO cells were incubated in either the presence or the absence of 1 ng/ml of TGF-β1 for the indicated time. *A*, light microscopic images. *B*, expression of E-cadherin detected by immunoblotting. α-Tubulin was used as a loading control. *C*, formation of actin stress fibers. F-actin was stained using rhodamine–phalloidin. *D*, immunofluorescence detection of E-cadherin. *E*, cell growth rates evaluated by cell number counting. TGF-β1 was added on day 1. *F*, chamber migration assay. TGF-β1 stimulation for 12 h. *G*, wound healing assay. TGF-β1 stimulation for 24 h. Quantification is shown on the *right*. Scale bars represent 50 μm (*A*, *C*, and *D*), 100 μm (*F*), and 200 μm (*G*). Error bars represent the SD (n = 5 for *F* and *G*). The *p* values were determined by Student’s *t* test. ∗*p* < 0.01. One representative result from two independent experiments is shown (*E*–*G*). TGF-β, transforming growth factor-β.

Although Smad2Δexon3 restored CAGA_12_-MLP-Luc reporter activity in A549-S3-KO cells, it remained unclear if other cellular responses, attenuated by Smad3 deficiency, could be restored. Therefore, as a next step, we examined cellular responses induced by TGF-β in A549-S2E3/S3-KO cells. We could observe that Smad2Δexon3 expression restored cell morphology changes, E-cadherin downregulation, stress fiber formation, and TGF-β–induced cytostasis (Fig. 2, *A*–*E*). However, we discovered that TGF-β–enhanced cell motility, assessed by chamber migration and wound healing assays, was not restored (Fig. 2, *F* and *G*). These findings indicate that Smad2Δexon3 has distinct signaling properties from Smad3.

### Both the Smad3 MH1 domain and linker region are required for the TGF-β–induced enhanced cell motility

**Figure 3:**
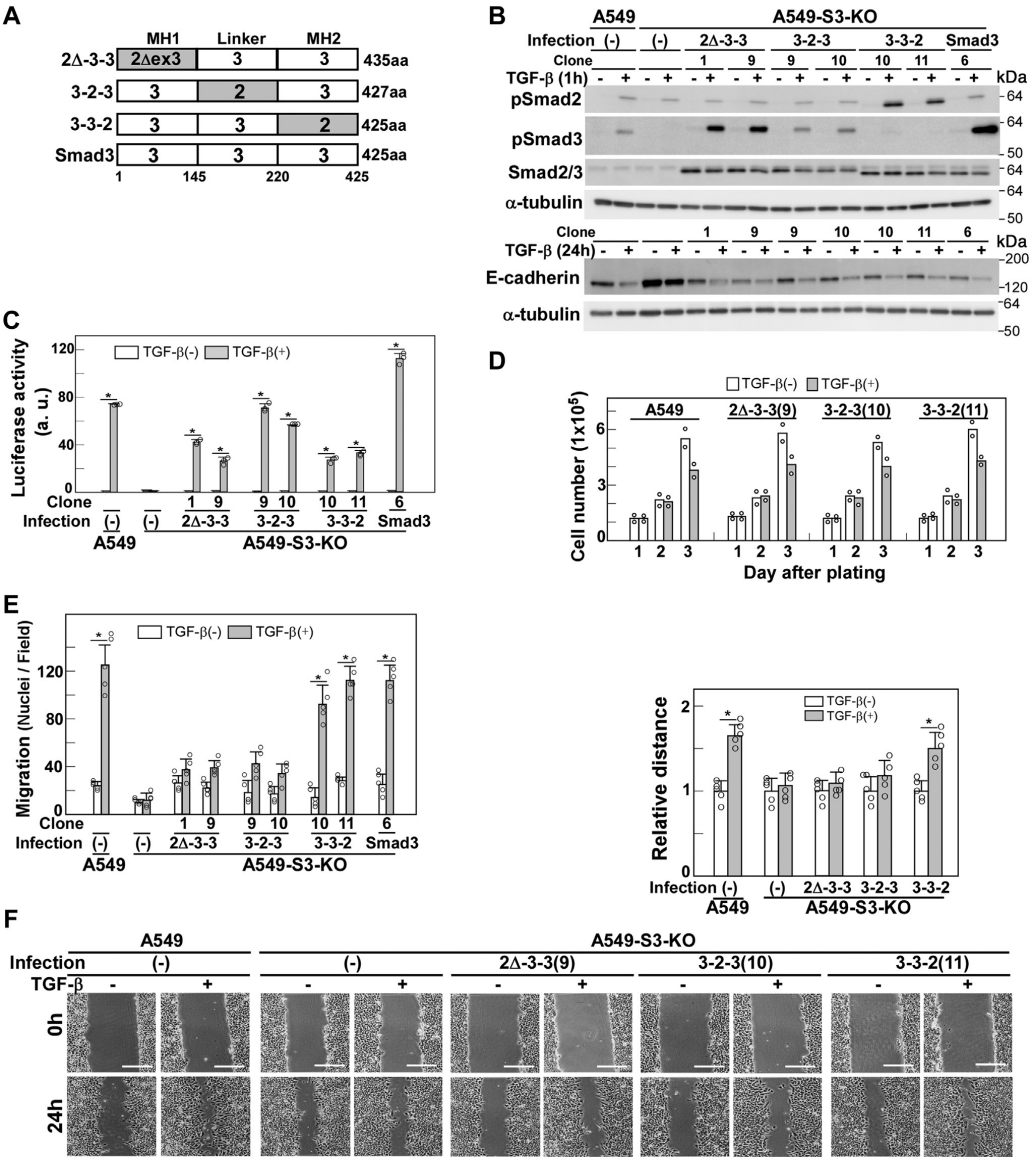
**Both the MH1 domain and linker region are necessary for TGF-β–enhanced cell motility.***A*, schematic presentation of the 2Δ-3-3, 3-2-3, and 3-3-2 chimeric proteins. 2Δ-3-3 (Smad3 with Met-1 to Thr-154 from Smad2Δexon3), 3-2-3 (Smad3 with Glu-185 to Leu-261 from Smad2), and 3-3-2 (Smad3 with Asp-262 to Ser-467 from Smad2). A549-S3-KO cells were infected with lentivirus carrying either the 2Δ-3-3, 3-2-3, or 3-3-2 chimera-encoding complementary DNA (cDNA). *B*–*F*, A549 cells, A549-S3-KO cells, or A549-S3-KO cells expressing either WT Smad3, 2Δ-3-3, 3-2-3, or 3-3-2 were incubated in either the presence or the absence of 1 ng/ml of TGF-β1 for the indicated time. *B*, the expression and TGF-β–induced phosphorylation of Smad2Δexon3/Smad3 chimeras were verified by immunoblotting with the indicated antibodies. The expression of E-cadherin was determined by immunoblotting with anti-E-cadherin; α-tubulin was used as a loading control. *C*, CAGA-Luc assay. TGF-β1 stimulation for 18 h. *D*, cell growth rate. *E*, chamber migration assay. TGF-β1 stimulation for 12 h. *F*, wound healing assay. TGF-β1 stimulation for 24 h. Quantification is shown at the *top*. The scale bars represent 200 μm (*F*). Error bars represent the SD (n = 3 for *C* and n = 5 for *E* and *F*). The *p* values were determined by Student’s *t* test. ∗*p* < 0.01. One representative result from two independent experiments is shown (*C–F*). TGF-β, transforming growth factor-β.

In order to identify the region in Smad2Δexon3 responsible for the failure in rescuing cell motility, we introduced chimeric Smad2Δexon3/Smad3 proteins into A549-S3-KO cells. These chimeric proteins, S2Δ/3(Δ2-3-3), S2Δ/3(3-2-3), and S2Δ/3(3-3-2), contained either the MH1, linker, or MH2 domains of Smad3, respectively, replaced by the corresponding region from Smad2Δexon3 (Fig. 3*A*). Cell clones expressing either of these chimeric proteins were isolated and selected for equivalent chimeric protein expression levels (Fig. 3*B*). We verified the C-terminal phosphorylation of these chimeric proteins in response to TGF-β, although their phosphorylation levels were lower than those of Smad3 (Fig. 3*B*). All three chimeric proteins rescued the CAGA_12_-MLP-Luc reporter activity (Fig. 3*C*), E-cadherin downregulation (Fig. 3*B*), and cytostasis (Fig. 3*D*) in response to TGF-β, indicating successful signal transduction. However, enhanced cell motility could be restored only by S2Δ/3(3-3-2) (Fig. 3, *E* and *F*). These results indicate that both the Smad3 MH1 domain and linker region are essential for signal transmission to enhance cell motility.

### Lysine-114 substitution with arginine in the β4 region renders the Smad2Δexon3 MH1 domain the signal transmission ability leading to enhanced cell motility

**Figure 4:**
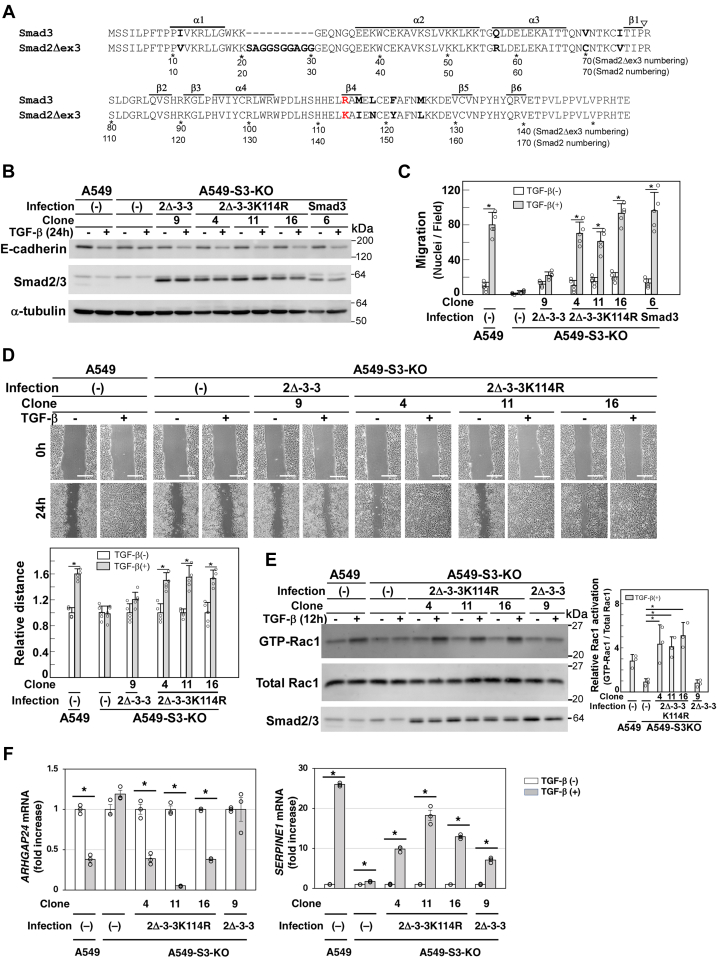
**Arginine substitution of Lys-114 increased the 2Δ-3-3 activity to mediate TGF-β*–*enhanced cell motility.***A*, alignment of the Smad2Δexon3 and Smad3 MH1 domain sequences. Diverged residues are shown in *bold*. *Arrowhead* indicates the position of exon 3. Arg-104 in Smad3 and Lys-114 in Smad2Δexon3 are shown in *red*. Lys-114 in Smad2Δexon3 was substituted by Arg (2Δ-3-3K114R). The A549-S3-KO cells were infected with lentivirus carrying 2Δ-3-3K114R chimera-encoding complementary DNA (cDNA). *B*–*F*, A549 cells, A549-S3-KO cells, or A549-S3-KO cells expressing either WT Smad3, 2Δ-3-3, or 2Δ-3-3K114R were incubated in either the presence or the absence of 1 ng/ml of TGF-β1 for the indicated time. *B*, the expressions of Smad2Δexon3/Smad3 chimeras and E-cadherin were determined by immunoblotting with the indicated antibodies; α-tubulin was used as a loading control. *C*, chamber migration assay. TGF-β1 stimulation for 12 h. *D,* wound healing assay. TGF-β1 stimulation for 24 h. Quantification is shown in the *bottom*. *E*, Rac1 activation assay. The amount of active GTP-loaded Rac1 was determined by using a glutathione-*S*-transferase (GST) pull-down assay. Rac1 was detected by immunoblotting. One representative result from three independent experiments is shown. Others are presented in Fig. S2. Quantification is shown on the *right*. *F*, TGF-β–induced *ARHGAP24* downregulation. Cells were stimulated with TGF-β1 for 8 h on collagen-coated plates and subjected to quantitative real-time PCR. The scale bars represent 200 μm (*D*). Error bars represent the SD (n = 3 for *E* and *F* and n = 5 for *C* and *D*). The *p* values were determined by Student’s *t* test (*C*, *D*, and *F*) or Dunnet’s multiple comparison test (*E*). ∗*p* < 0.01. One representative result from two independent experiments is shown (*C*, *D*, and *F*). TGF-β, transforming growth factor-β.

The MH1 domains of Smad2Δexon3 and Smad3 share 88% identity with an insertion of 10 additional amino acid residues in Smad2 (Fig. 4*A*). We recently reported that the β4 region in the Smad3 MH1 domain is crucial for cell motility–related signal transmission, *via* downregulating *ARHGAP24* to prevent accelerated Rac1 inactivation (25). A divergent residue could be identified between Smad2Δexon3 (Lys-114) and Smad3 (Arg-104) in this region (Fig. 4*A*). Therefore, we constructed a Lys114Arg mutant of the S2Δ/3(Δ2-3-3) chimeric protein and introduced it into A549-S3-KO cells (Fig. 4*B*). As anticipated, TGF-β–enhanced cell motility, Rac1 activation, and downregulation of *ARHGAP24* were successfully restored by this mutant (Fig. 4, *C*–*F*).

### The linker region Asn-210 in Smad3 is required for the TGF-β–induced enhanced cell motility

**Figure 5:**
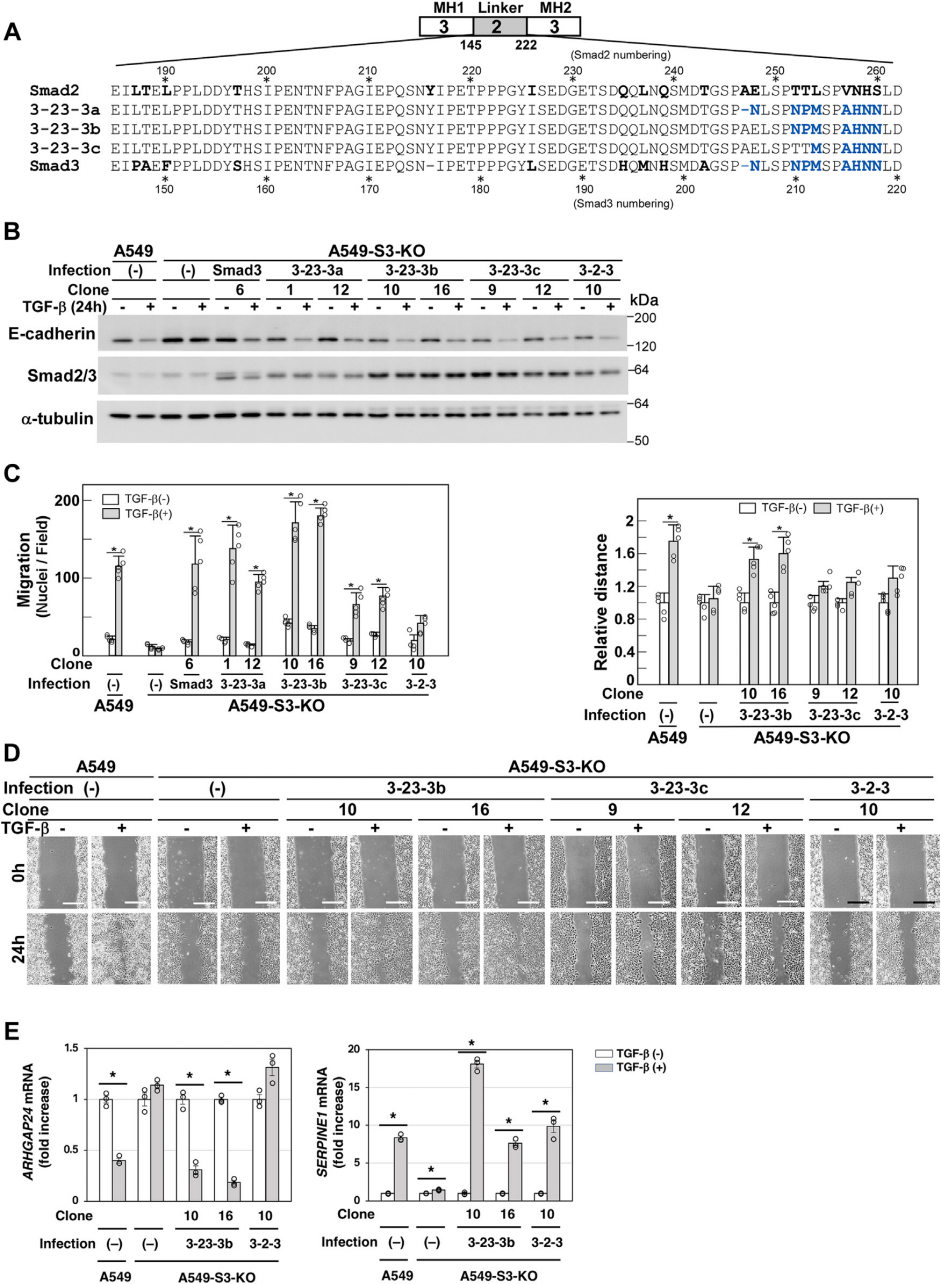
**The C-terminal region of the linker in Smad3 is involved in TGF-β–induced downregulation of *ARHGAP24*.***A*, schematic presentation of 3-23-3 chimeric proteins. 3-2-3 (Smad3 with Glu-185 to Leu-261 from Smad2); 3-23-3a (Smad3 with Glu-185 to Pro-246 from Smad2); 3-23-3b (Smad3 with Glu-185 to Pro-251 from Smad2); and 3-23-3c (Smad3 with Glu-185 to Thr-253 from Smad2). A549-S3-KO cells were infected with lentivirus carrying complementary DNA (cDNA) encoding either the 3-23-3a, 3-23-3b, or 3-23-3c chimera. Diverged residues between Smad2 and Smad3 are shown in *bold*. Residues derived from Smad3 are shown in *blue*. *B*–*E*, A549 cells, A549-S3-KO cells, or A549-S3-KO cells expressing either WT Smad3, 3-2-3, 3-23-3a, 3-23-3b, or 3-23-3c were incubated in either the presence or the absence of 1 ng/ml TGF-β1 for indicated time. *B*, expression of Smad2Δexon3/Smad3 chimeras and E-cadherin was determined by immunoblotting with the indicated antibodies; α-tubulin was used as a loading control. *C*, chamber migration assay. TGF-β1 stimulation for 12 h. *D,* wound healing assay. TGF-β1 stimulation for 24 h. Quantification is shown at the *top*. *E,* TGF-β–induced downregulation of *ARHGAP24*. Cells were stimulated with TGF-β1 for 8 h on collagen-coated plates and subjected to quantitative real-time PCR. The scale bars represent 200 μm (*D*). Error bars represent SD (n = 3 for *E* and n = 5 for *C* and *D*). The *p* values were determined by Student’s *t* test. ∗*p* < 0.01. One representative result from two independent experiments is shown (*C–E*). TGF-β, transforming growth factor-β.

Next, we aimed to narrow down the linker region responsible for the enhanced cell motility. The linker regions of Smad2 and Smad3 display diverged residues in the C-terminal subregion (residues Ala-247–Ser-260 in Smad2 and Asn-206–Asn-218 in Smad3) (Fig. 5*A*). We constructed a series of S2Δ/3(3-2-3) chimeric proteins with replacement to corresponding residues in Smad3; S2Δ/3(3-23-3a), 206 to 218 in Smad3; S2Δ/3(3-23-3b), 210 to 218 in Smad3; and S2Δ/3(3-23-3c), 212 to 218 in Smad3. A549-S3-KO cells were infected with complementary DNAs (cDNAs) encoding these chimeric proteins. All these chimeric proteins rescued E-cadherin downregulation (Fig. 5*B*). Moreover, S2Δ/3(3-23-3a) and S2Δ/3(3-23-3b) restored TGF-β–enhanced cell motility, although we could observe only a modest effect in the case of S2Δ/3(3-23-3c) (Fig. 5*C*). We verified that S2Δ/3(3-23-3b) also restored cell motility in the wound healing assay (Fig. 5*D*). Therefore, residues Asn-210–Asn-218 in Smad3, corresponding to Thr-252–Ser-260 in Smad2, play a role in signal transmission to enhance cell motility. Notably, S2Δ/3(3-23-3b), but not S2Δ/3(3-2-3), could downregulate *ARHGAP24* after TGF-β stimulation (Fig. 5*E*), suggesting that the downregulation is essential for enhancing cell motility.

**Figure 6:**
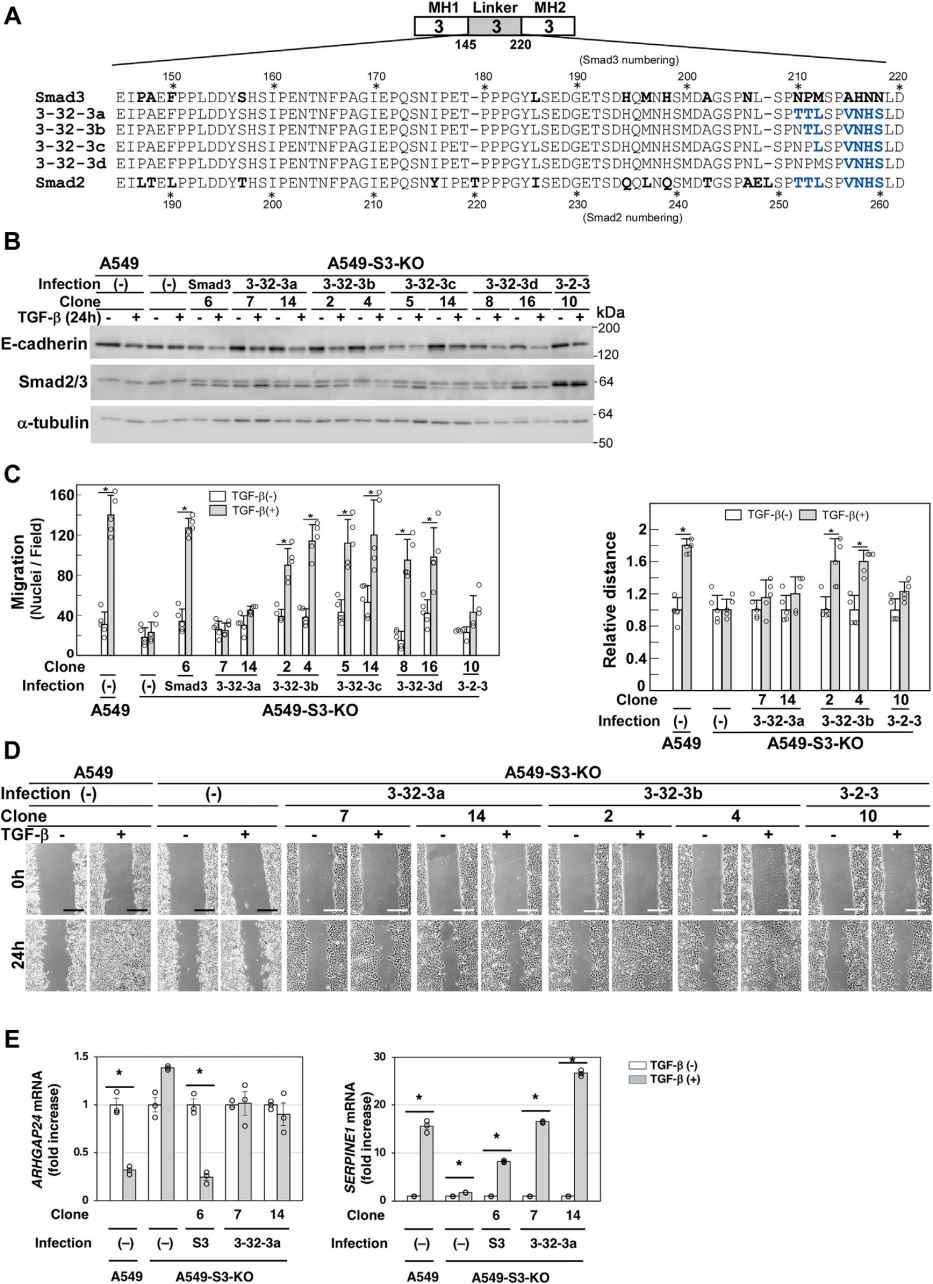
**Asn-210 in Smad3 is involved in TGF-β–induced downregulation of *ARHGAP24*.***A,* schematic presentation of 3-32-3 chimeric proteins. 3-32-3a (Smad3 with Thr-252 to Ser-260 from Smad2); 3-32-3b (Smad3 with Thr-253 to Ser-260 from Smad2); 3-32-3c (Smad3 with Leu-254 to Ser-260 from Smad2); 3-32-3d (Smad3 with Ser-255 to Ser-260 from Smad2). A549-S3-KO cells were infected with lentivirus carrying complementary DNA (cDNA) encoding either the 3-32-3a, 3-32-3b, 3-32-3c, or 3-32-3d chimera. Diverged residues between Smad2 and Smad3 are shown in *bold*. Residues derived from Smad2 are shown in *blue*. *B*–*E*, A549 cells, A549-S3-KO cells, or A549-S3-KO cells expressing either WT Smad3, 3-2-3, 3-32-3a, 3-32-3b, 3-32-3c, or 3-32-3d were incubated in either the presence or the absence of 1 ng/ml TGF-β1 for indicated time. *B*, expression of Smad2Δexon3/Smad3 chimeras and E-cadherin was determined by immunoblotting with the indicated antibodies. α-tubulin was used as a loading control. *C*, chamber migration assay. TGF-β1 stimulation for 12 h. *D*, wound healing assay. TGF-β1 stimulation for 24 h. Quantification is shown at the *top*. *E*, TGF-β–induced downregulation of *ARHGAP24*. Cells were stimulated with TGF-β1 for 8 h on collagen-coated plates and subjected to quantitative real-time PCR. The scale bars represent 200 μm (*D*). Error bars represent SD (n = 3 for *E* and n = 5 for *C* and *D*). The *p* values were determined by Student’s *t* test. ∗*p* < 0.01. One representative result from two independent experiments is shown (*C–E*). TGF-β, transforming growth factor-β.

We wanted to further identify amino acid residues crucial for cell motility in the linker region. We thus constructed S2Δ/3(3-32-3a), in which the C-terminal stretch of the amino acid residues Asn-210–Asn-218 in Smad3 was substituted with Thr-252–Ser-260 in Smad2 (Fig. 6*A*). In addition, we constructed three additional chimeric proteins S2Δ/3(3-32-3b), S2Δ/3(3-32-3c), and S2Δ/3(3-32-3d) with substituted residues replaced one by one with the corresponding Smad3 residues (Fig. 6*A*). We introduced these constructs into A549-S3-KO cells and selected stably expressing clones. All these chimeric proteins rescued TGF-β–induced E-cadherin downregulation (Fig. 6*B*), but only S2Δ/3(3-32-3a) failed to enhance cell motility in the chamber migration and wound healing assays (Fig. 6, *C* and *D*). As S2Δ/3(3-32-3a) and S2Δ/3(3-32-3b) differ by only a single amino acid residue, Asn-210 in Smad3 and Thr-252 in Smad2 (Fig. 6*A*), that residue is crucial for exerting TGF-β–induced enhanced cell motility. In addition, S2Δ/3(3-32-3a), which failed to rescue TGF-β–enhanced cell motility, did not transmit signals to downregulate *ARHGAP24* (Fig. 6*E*). Therefore, the C-terminal stretch of the linker region appears to be involved in *ARHGAP24* downregulation and subsequent cell motility enhancement.

### Smad2Δexon3 acquires ability to enhance cell motility by mutation of two amino acid residues, Lys-114 and Thr-222, to corresponding residues in Smad3

**Figure 7:**
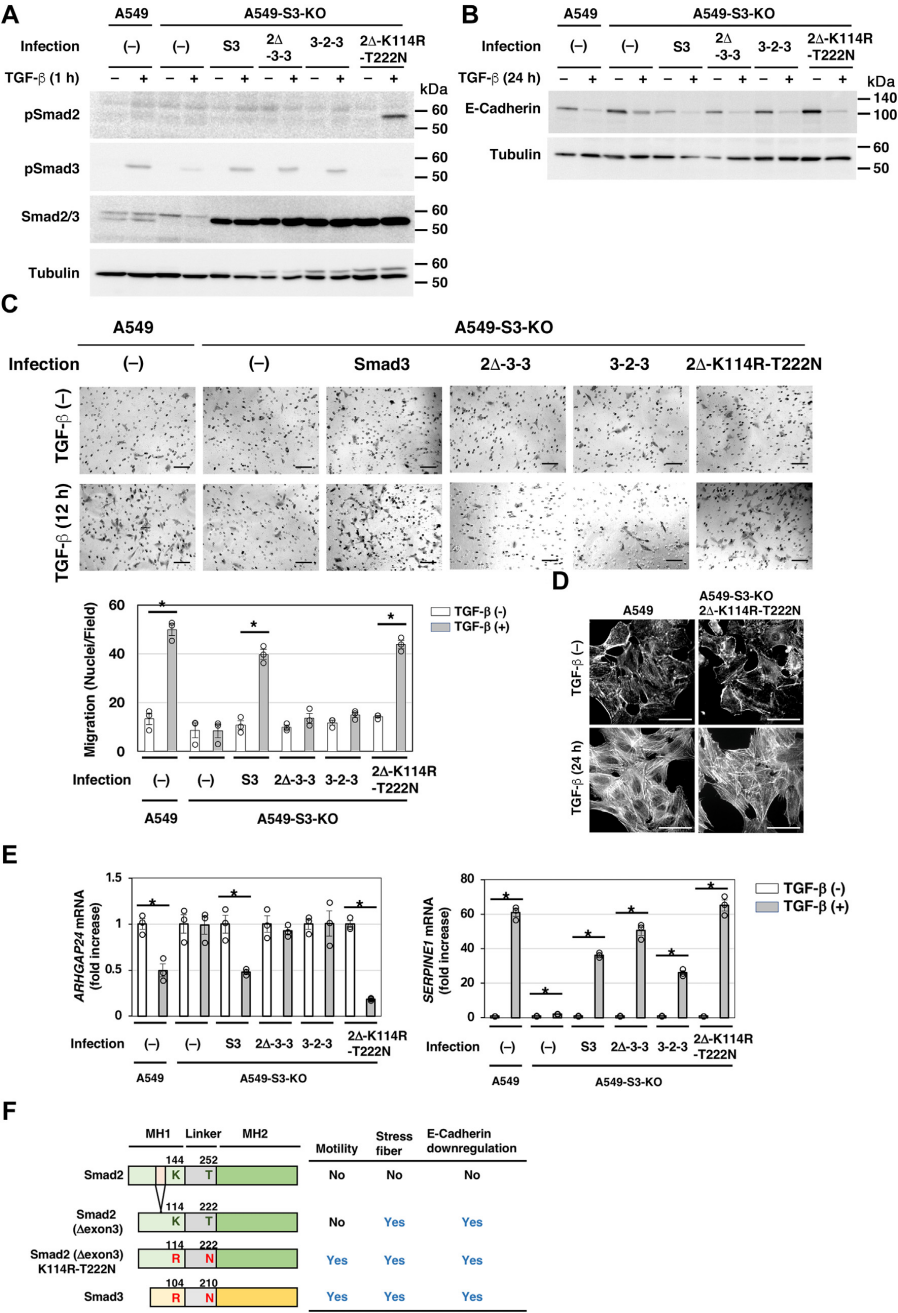
**Lys114Arg and Thr222Asn substitutions increased the Smad2**Δ**exon3 activity to mediate TGF-β-enhanced cell motility.** A549-S3-KO cells were infected with lentivirus carrying complementary DNA (cDNA) encoding Smad2Δexon3 with Lys114Arg and Thr222Asn substitutions (2Δ-K114R-T222N), Smad3, chimeric proteins 2Δ-3-3 or 3-2-3, and used as cell pools. *A*, A549 cells, A549-S3-KO cells, or A549-S3-KO cells expressing either WT Smad3 (S3), 2Δ-3-3, 3-2-3, or 2Δ-K114R-T222N were incubated in either the presence or the absence of 1 ng/ml of TGF-β1 for the indicated time. The expression and TGF-β–induced phosphorylation of Smad2Δexon3/Smad3 chimeras and the Smad2Δexon3 amino acid substitution mutant were verified by immunoblotting with the indicated antibodies. *B*, the expression of E-cadherin was determined by immunoblotting with anti-E-cadherin; α-tubulin was used as a loading control. *C*, chamber migration assay. TGF-β1 stimulation for 12 h. Quantification is shown in the *bottom*. *D*, formation of actin stress fibers. F-actin was stained using rhodamine–phalloidin. *E*, cells were stimulated with TGF-β1 for 8 h on collagen-coated plates and subjected to quantitative real-time PCR. The scale bars represent 100 μm (*C*) and 25 μm (*D*). Error bars represent SD (n = 3 for *D* and n = 4 for *C*). The *p* values were determined by Student’s *t* test. ∗*p* < 0.01. One representative result from two independent experiments is shown (*C* and *D*). *F*, schematic illustration of differences among Smad2, Smad2Δexon3, and Smad3 in TGF-β signaling. Smad2 and Smad3 differ in their activities in TGF-β–induced cellular responses. Smad2Δexon3 transmits Smad3-dependent cellular responses other than cell motility. Arg-104 and Asn-210 in Smad3 contribute to TGF-β–enhanced cell motility through the downregulation of *ARHGAP24*. TGF-β, transforming growth factor-β.

In order to confirm the importance of two amino acid residues, Arg-104 and Asn-210, in Smad3, we also constructed a double mutant Smad2Δexon3 (Lys114Arg/Thr222Asn) and introduced it into A549-S3KO cells (Fig. 7*A*). We found that the mutant can rescue TGF-β–induced E-cadherin downregulation (Fig. 7*B*), cell motility (Fig. 7*C*), actin stress fiber formation (Fig. 7*D*), and *ARHGAP24* downregulation (Fig. 7*E*), indicating that Smad2Δexon3 acquires ability to enhance cell motility by mutation in only two amino acid residues. Figure 7*F* summarizes Smad2, Smad2Δexon3, Smad2Δexon3 (Lys114Arg/Thr222Asn), and Smad3 ability to support EMT-associated cellular responses.

### Knockdown of ARHGAP24 rescued the defect of TGF-β–enhanced cell motility in A549-S2E3/S3-KO cells

**Figure 8:**
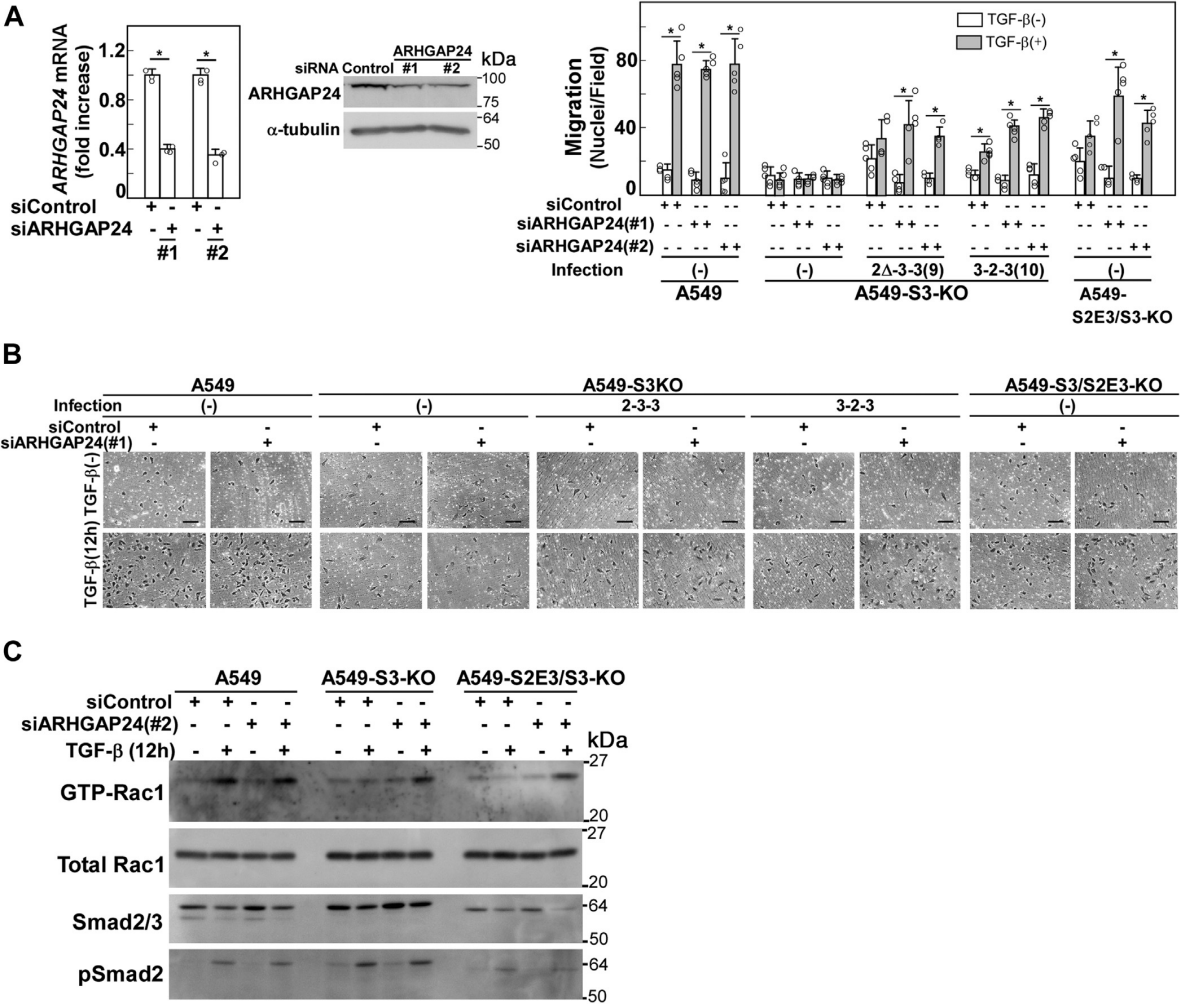
**Knockdown of *ARHGAP24* rescues the defect of TGF-β–enhanced cell motility in A549-S2E3/S3-KO cells**. *A*, knockdown of *ARHGAP24* in A549 cells. Cells were treated with control siRNA (siControl) or *ARHGAP24* siRNA for 24 h. The expression levels of mRNA and protein were measured by quantitative real-time PCR and immunoblotting, respectively. *B*, effect of *ARHGAP24* knockdown on TGF-β–enhanced cell motility in A549-S3-KO cells expressing Smad2Δexon3/Smad3 chimeras or A549-S2E3/S3-KO cells. Cells were treated with 5 nM siControl or siARHGAP24 for 24 h and harvested for chamber migration assay (TGF-β1 stimulation for 12 h). The scale bars represent 100 μm. Quantification is shown at the *top*. Error bars represent SD (n = 5). The *p* values were determined by Student’s *t* test. ∗*p* < 0.01. *C*, Rac1 activation assay. The amount of active GTP-loaded Rac1 was determined by using a glutathione-*S*-transferase (GST) pull-down assay. Rac1 was detected by immunoblotting. One representative result from two independent experiments is shown (*A*–*C*). TGF-β, transforming growth factor-β.

Finally, we examined how *ARHGAP24* knockdown could affect TGF-β–enhanced cell motility. *ARHGAP24* knockdown was verified by mRNA and protein expression analyses (Fig. 8*A*). Figure 3*E* shows that TGF-β failed to enhance cell motility in A549-S3-KO cells expressing S2Δ/3(3-2-3) or S2Δ/3(Δ2-3-3), whereas TGF-β successfully enhanced cell motility upon *ARHGAP24* knockdown in these cells (Fig. 8*B*). These findings indicate that *ARHGAP24* downregulation is the key biochemical event downstream of the Smad3 MH1 domain and linker region during TGF-β–mediated cell motility enhancement. However, these regions might induce other biochemical events leading to enhanced cell motility to some extent, as the *ARHGAP24* knockdown–induced cell motility restoration was partial.

Moreover, we examined TGF-β–induced Rac1 activation after *ARHGAP24* knockdown (Fig. 8*C*). In parental A549 cells, TGF-β–induced Rac1 activation was not affected by *ARHGAP24* knockdown. However, TGF-β activated Rac1 and enhanced cell motility only after the *ARHGAP24* knockdown in A549-S2E3/S3-KO cells. Therefore, A549-S2E3/S3-KO cells expressing only Smad2Δexon3 as a TGF-β signal–mediating R-Smad protein fail to enhance cell motility because of deficient Rac1 activation. Interestingly, TGF-β activated Rac1 *via* the PI3K pathway but failed to enhance cell motility in A549-S3-KO cells upon *ARHGAP24* knockdown (Fig. 8, *B* and *C*, also shown in Ref. (25)). These findings indicate that Smad3 also transmits signals unrelated to Rac1 activation.

## Discussion

Smad proteins play a crucial role in signal transduction downstream of cytokines of the TGF-β family. Among those, Smad2 and Smad3 principally mediate TGF-β-, activin-, and Nodal-related signaling to the nucleus. Although these two Smad proteins share 92% sequence similarity, their functions have been considered distinct (6, 26, 27, 28). TGF-β–induced growth inhibition of epithelial cells is dependent on Smad3 but not Smad2 (29). Breast cancer cells become more aggressive in the absence of Smad2 in part because Smad2 represses basal vascular endothelial growth factor A expression (15). Certain TGF-β–induced genes are both Smad2 and Smad3 dependent, whereas others solely depend either on Smasd2 or Smad3 (16, 28). In certain cases, Smad2 and Smad3 counteract each other (8, 10, 11, 12, 15). However, the differences in Smad2 and Smad3 signaling properties have not yet been well understood. In the present study, we described the distinct cell signaling properties of Smad2Δexon3, an alternatively spliced Smad2 isoform, and Smad3.

### Function of the Smad2Δexon3 isoform

Although Smad2Δexon3 is a minor Smad2 isoform in mouse tissues (9), its signaling properties during embryonic development have already been examined (23). Gene-engineered mice exclusively expressing Smad2Δexon3 as R-Smad downstream of TGF-β/activin/Nodal (*Smad2*^*Δexon3*^*:Smad3*^*null*^ homozygous mice) exhibit no apparent phenotypes except for osteoarthritis, observed in *Smad3*^*null*^ mice. In addition, *Smad2*^*null*^ homozygous mice expressing Smad2Δexon3 are viable, whereas those expressing the full-length Smad2 are embryonic lethal (23). Therefore, Smad2Δexon3-mediated signaling is necessary and sufficient for embryonic development.

In contrast, Smad2Δexon3 function in adult tissue homeostasis remains elusive. In several healthy and cancerous human cell lines, Smad2Δexon3 represents only a small fraction of the total Smad2 protein (19). To date, several Smad3-specific binding partners have been identified (6, 30). However, certain Smad3-specific binding partners reportedly interact with Smad2Δexon3 (6). Therefore, it was anticipated that certain defects caused by the loss of Smad3 could be restored by Smad2Δexon3. Consistently, we found that Smad2Δexon3 rescued multiple TGF-β–induced EMT-related cellular phenotypes but not enhanced cell motility. Together with the previous finding that Smad2Δexon3 is sufficient for embryonic development (23), TGF-β/activin/Nodal-induced cell motility might be dispensable for morphogenesis.

### The underlying mechanisms of TGF-β–enhanced cell motility through Smad3

TGF-β is a representative extracellular EMT inducer. We previously reported that signals leading to cell motility are transmitted independently of those leading to other EMT-associated cell responses (25, 31, 32). TGF-β–induced cell motility is thought to contribute to the invasive and metastatic properties of malignant tumor cells. Consistently, blocking Smad signaling reportedly attenuates the metastasis of breast cancer cells without affecting primary tumor growth (33). Thus far, several Smad-binding transcription factors (Smad cofactors), such as JunB (34), Olig1 (31), and Sox4 (35), have been reportedly involved in TGF-β–induced cell motility or invasive properties. In addition, Smad3 linker phosphorylation reportedly enhanced cell motility *via* a Pin1-dependent mechanism in PC-3 human prostate cancer cells and normal murine mammary gland epithelial cells (31, 36). However, the downstream events leading to enhanced cell motility remained poorly understood.

We recently reported that the Smad3 β4 region in the MH1 domain is involved in transmitting signals triggering cell motility. The signal through the β4 region leads to *ARHGAP24* (encoding Fil-GAP, a Rac1-targeting GAP) downregulation, a key event during TGF-β–enhanced cell motility, although this is not the sole Smad3-mediated signaling output (25). In this study, we found that the Smad3 linker region is also required for downregulating *ARHGAP24*.

Any potential cooperativity between the β4 and the linker regions in downregulating *ARHGAP24* remains to be elucidated. Alternatively, these two regions might independently affect *ARHGAP24* downregulation. Intriguingly, the responsible region in the linker (Asn-210–Asn-218 in Smad3 and Thr-252–Ser-260 in Smad2) is closely located or overlapped with the linker phosphorylation sites (37). In the C-terminal half of the linker region, Smad3 displays three phosphorylation sites (Ser-204, Ser-208, and Ser-213), whereas Smad2 exhibits six phosphorylation sites (Ser-240, Ser-245, Ser-250, Thr-252, Ser-255, and Ser-260). Among these Smad2 sites, four (Ser-240, Ser-245, Ser-250, and Ser-255) are shared with Smad3, whereas Thr-252 and Ser-260 are not conserved between Smad2 and Smad3 (Fig. 5*A*). Thr-252 and Ser-260 are phosphorylated by Araf kinase (38) and calmodulin-dependent kinase II (39), respectively. A possibility is that Thr-252 (Thr-222 in Smad2Δexon3) phosphorylation is inhibitory to the linker-mediated signaling through attenuating or facilitating the physical interaction with certain binding partners. However, this possibility could be excluded because when Asn-210 in Smad3 was mutated to Ala, TGF-β–enhanced cell motility was attenuated (Fig. S3). Therefore, Asn-210 is likely to play an enhancing role in signal transmission. We previously found that the presence of the linker region affects the MH2 domain–mediated interaction with Olig1 (31). Therefore, the linker sequence might affect the MH2 domain–mediated physical interaction. Arg-104- and Asn-210-dependent Smad3 binding partners, which are likely to be involved in enhanced cell motility, remain to be identified.

### Future perspectives

We previously reported that TGF-β–induced actin stress fiber formation and cell morphological changes are also dependent on Smad3 (25). We have previously attempted to identify the region in Smad3 responsible for the signaling inducing these effects through approaches using Smad1/Smad3 chimeric proteins, although we could not obtain satisfying results (25). However, we recently found that these responses are dependent on Snail, a core EMT-transcription factor (K. Miyazawa, unpublished observations). Snail can be induced by both TGF-β- and bone morphogenetic protein–related signaling (40), the latter of which use Smad1, Smad5, and Smad8 as downstream effectors. Therefore, approaches using Smad1/3 chimeric proteins might not be appropriate for identifying the region in Smad3 required for actin stress fiber formation and cell morphological changes. Intriguingly, Snail knockdown also attenuated TGF-β–enhanced cell motility (K. Miyazawa, unpublished observations). We previously reported that Smad3 exhibits a function other than downregulating *ARHGAP24* in enhanced cell motility (25). Therefore, the additional Smad3-mediated signaling might be associated with the Snail pathway, which appears to be common with Smad2Δexon3. Future investigation should focus on unveiling these unsolved questions.

## Experimental procedures

### Cell lines and CRISPR–Cas9 system-mediated genome editing

A549 cells were obtained from the American Type Culture Collection (41), cloned, and used as parental cells, authenticated by short tandem repeat analysis (4). The establishment of *SMAD3* KO A549 (A549-S3-KO) cells was described previously (4). The KO of *SMAD2* exon 3 was conducted using Double Nickase Plasmid (catalog no.: sc-400475-NIC-2; Santa Cruz Biotechnology). Deletion/disruption of the target exon was confirmed by sequencing (Fig. S1). The KO of exon 3 resulted in the dominant expression of Smad2Δexon3, an alternatively spliced isoform (Fig. 1*A*). Cells were maintained in Dulbecco’s modified Eagle's medium containing 10% fetal bovine serum, supplemented with 50 units/ml of penicillin and 50 μg/ml of streptomycin, at 37 °C under a 5% CO_2_ atmosphere.

### Reagents and antibodies

Human recombinant TGF-β1 was obtained from R&D Systems and used at 1 ng/ml unless otherwise indicated. Antibodies used were as follows: anti-Smad2/3 (Clone 18; BD Bioscience), anti-phosho-Smad2 (134D4, Cell Signaling Technology or A5S, Millipore), anti-phosho-Smad3 (C25A9, Cell Signaling Technology), anti-Smad4 (D3R4N, Cell Signaling Technology), anti-E-cadherin (Clone 36, BD Bioscience), anti-Rac1 (23A8, Millipore), anti-ARHGAP24 (ab203874, Abcam), and anti-α-tubulin (DM1A, Sigma–Aldrich). Horseradish peroxidase–conjugated goat anti-mouse immunoglobulin G (catalog no.: 115-035-003) and goat anti-rabbit immunoglobulin G (catalog no.: 1110035-003) (Jackson Immuno Research) were used as secondary antibodies.

### Biochemical assays

Cell lysis and immunoblotting were performed as described previously using semidry transfer (31) except for Figure 1, in which wet tank transfer was used to detect lowly expressed endogenous Smad3. Immunofluorescent E-cadherin detection was described previously (42). Actin stress fiber formation was detected by rhodamine-conjugated phalloidin staining (Cytoskelton, Inc) as described previously (43). Rac1 activation, chamber migration, wound healing, and cell proliferation assays were performed as described previously (25, 32). The luciferase reporter assay, DNA affinity precipitation assay using 3xCAGA probe, and quantitative real-time PCR were performed as described previously (44). Table S1 shows the primer sequences for quantitative real-time PCR.

### Conventional RT–PCR

To detect Smad2 isoform expressions, RT–PCR analysis was performed. Total RNAs were isolated by using ISOGEN (NIPPON GENE). First-strand cDNA synthesis was performed using the PrimeScript first Strand cDNA Synthesis Kit and random hexamers (Takara Bio). The sequences of the applied primers were 5′-TTTTCCTAGCGTGGCTTG (forward) and 5′-CTGGTGTCTCAACTCTCTGA (reverse) (19).

### Lentivirus infection

The lentiviral vectors encoding either Smad3 or Smad2/3 chimeras were generated by the Gateway technology (Invitrogen). Lentivirus particles were produced as previously described (41) and then used to infect A549-S3-KO cells. Cells infected with Smad3 or Smad2/3 chimeras were cloned by limiting dilution in 96-well plates. Multiple clones with equivalent infected protein expression levels were used for the experiments. For experiments in Figure 7, pools of infected cells were used.

### RNAi

siRNAs were transfected into the cells (2 × 10^5^) using the Lipofectamine RNAiMAX transfection reagent (Invivogen). The stealth RNAi siRNAs against human *ARHGAP24* (#1, 5′-AAGAUAGAGUAUGAGUCCAG-GAUAA-3′; #2, 5′-CAGUGGUAAAUUAC-AACCUCCUCAA-3′) and siRNA Negative Control low GC Duplex were obtained from Invitrogen.

### Statistical analysis

A two-sided Student’s *t* test or Dunnet’s multiple comparison test was used to determine the significant differences among the experimental groups. Probability values below 0.01 were considered significant; ∗*p* < 0.01.

## Data availability

Data are contained within the article and its supporting information.

## Supporting information

This article contains supporting information (Table S1 and Figs. S1–S3).

## Conflict of interest

The authors declare that they have no conflicts of interest with the content of this article.
