# Supplementary material for: Smad2Δexon3 and Smad3 have distinct properties in signal transmission leading to TGF-β–induced cell motility
Source: J Biol Chem. 2022 Dec 20;299(2):102820. doi: 10.1016/j.jbc.2022.102820 (PMC9852702; doi:10.1016/j.jbc.2022.102820)
Supplement: Supporting information [file mmc1.pdf]

Supporting Information for

Smad2Δexon3 and Smad3 have distinct properties in signal transmission leading to TGF-β–induced cell motility

Takashi Yokoyama, Takahito Kuga, Yuka Itoh, Shigeo Otake, Chiho Omata, Masao Saitoh, and Keiji Miyazawa

Supplementary Figures: Fig. S1, S2, and S3

Supplementary Table: Table S1

Fig.S1

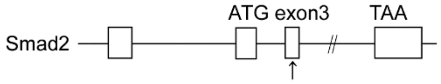


```
Wild type  TTCACCTGGTTTGTTCAGAGAAGCTGTAAAGGCCTGTTGTATCCCACTGATCTATCGTAT
Allele #1  TTCACCTGGTTTGTTCAGAGAAGCTGTAAAGGCCTGTTG-----------------GTAT -17
Allele #2  TTCA(                 )TGTAAAGGCCTGTTGTATCCCACTGATCTATCGTAT +947

(    ): ACAGGCCTTTCAGGTCGCCCTCGATCAGGAAGTGGCCCCGGAACTTGATCATGTGGGCCAGGGCCAGATAGATCAGCCGCAGGTCGGCCTT
GTCGGTGCTGTCCACCAGTTTCTTTCTCAGGTGGTAGATGGTGGGGTACTTCTCGTGGTAGGCCACCTCGTCCACGATGTTGCCGAAGATGGGGTGCCG
CTCGTGCTTCTTATCCTCTTCCACCAGGAAGGACTCTTCCAGTCTGTGGAAGAAGCTGTCGTCCACCTTGGCCATCTCGTTGCTGAAGATCTCTTGCAG
ATAGCAGATCCCGGTTCTTCCGTCTGGTGTACCTTCTTCTGGCGGTTCTCTTCAGCCGGGTGGCCTCGGCTGTTTCGCCGCTGTCGAACAGCAGGGCTC
CGATCAGGTTCTTCTTGATGCTGTGCCGGTCGGTGTTGCCCAGCACCTTGAATTTCTTGCTGGGCACCTTGTACTCGTCGGTGATCACGGCCCAGCCCA
CAGAGTTGGTGCCGACTATCATATGCTTACCGTAACTTGAAAGTATTTCGATTTCTTGGCTTTATATATCTTGTGGAAAGGACGAAACACCGACAGCTT
CTCTGAACAAACCGTTTTAGAGCTAGAAATAGCAAGTTAAAATAAGGCTAGTCCGTTATCAACTTGAAAAAGTGGCACCGAGTCGGTGCTTTTTTGTTT
TAGAGCTAGAAATAGCAAGTTAAAATAAGGCTAGTCCGTTTTTAGCGTGCGCCAATTCTGCAGACAAATGGCTCTAGAGGTACCCGTTACATAACTT
ACGGTAAATGGCCCGCCTGGCTGACCG
```

Figure. S1. Genomic DNA sequences of the *SMAD2* allele in *SMAD2 exon 3*/*SMAD3*-double knockout A549 cells. *SMAD2 exon 3*/*SMAD3*-double knockout A549 cells (A549-S2E3/S3-KO) were prepared from *SMAD3* knockout A549 cells (A549-S3-KO) using CRISPR/Cas9-mediated genome editing. The two Smad2 alleles in A549-S2E3/S3-KO cells contain 17 bp-deletion or 947 bp-insertion in the exon3 of *SMAD2* gene as shown in allele #1 and #2.

Fig.S2

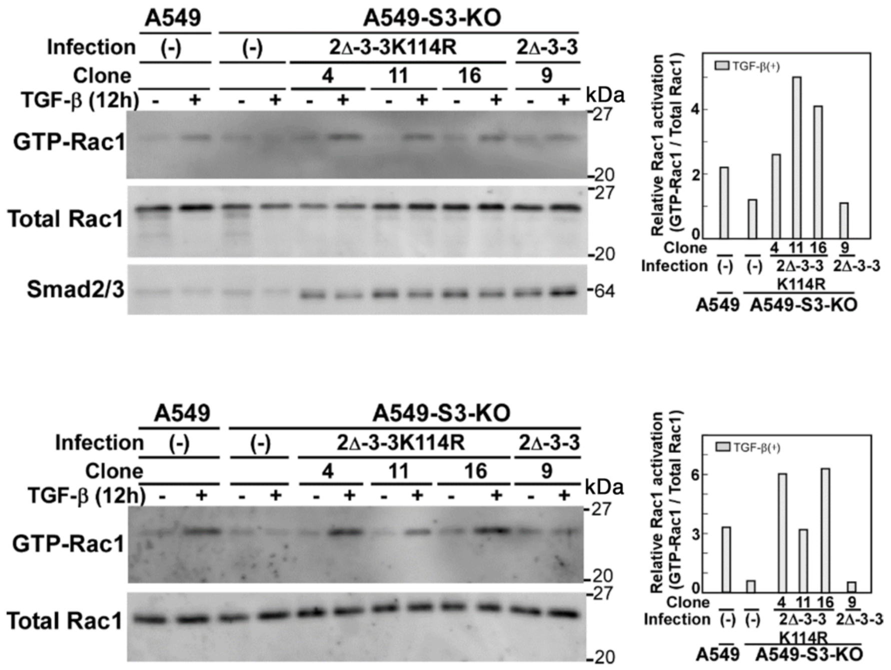

Figure. S2. Rac1 activation by TGF-β is restored by introduction of Smad2Δ3-3 K114R into A549-S3KO cells. Cells were subjected to Rac1 activation assay as in Fig. 4E. Quantification is shown on the *right*.

Fig.S3

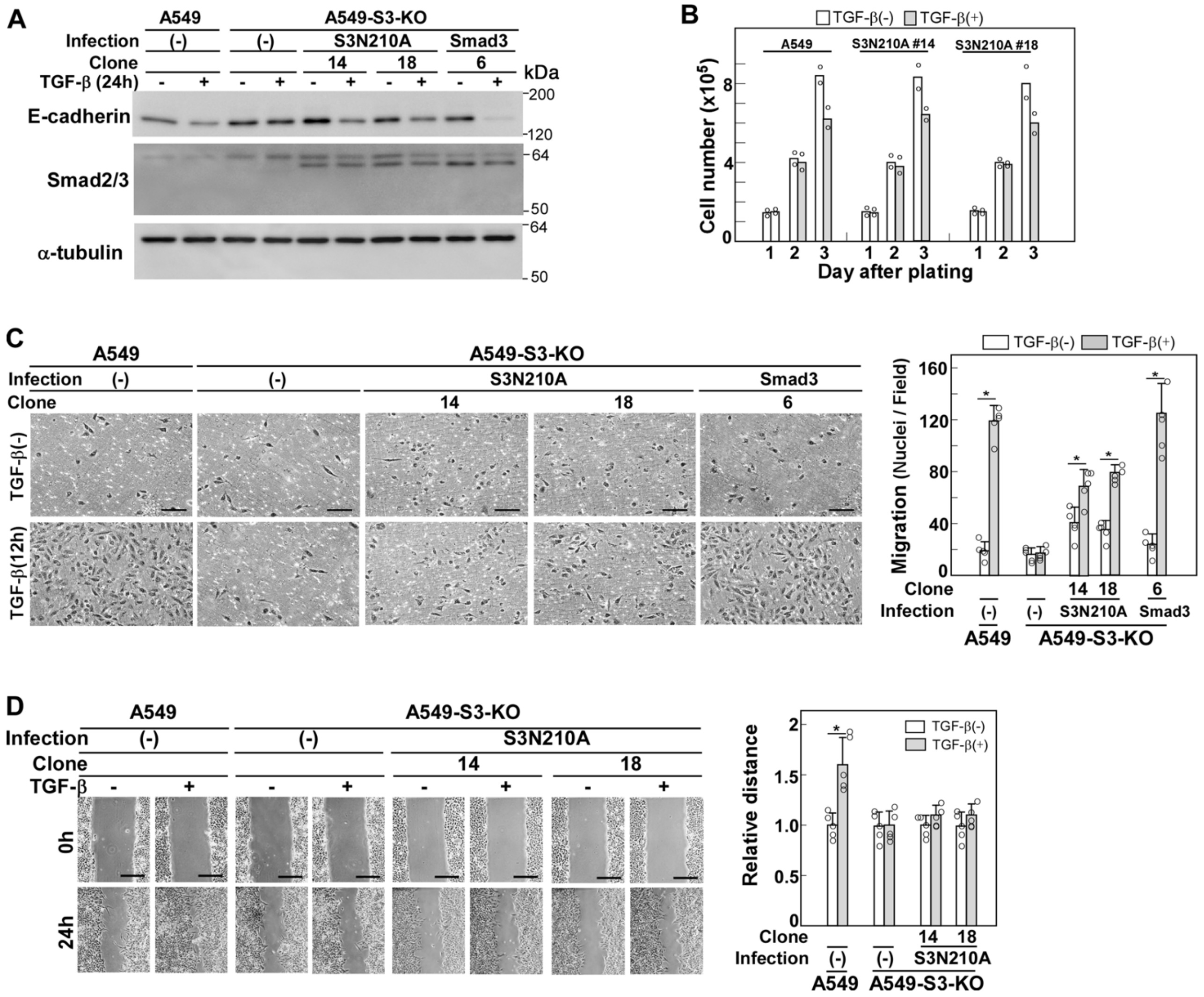

Figure. S3. Substitution of Asn-210 by Alanine attenuates the activity of Smad3 to mediate TGF-β–enhanced cell motility. A549-S3-KO cells were infected with lentivirus carrying cDNA encoding Smad3 with an Asn210Ala substitution (S3N210A). *A*, Expression of S3N210A and E-cadherin was determined by immunoblotting with the indicated antibodies; α-tubulin was used as a loading control. *B*, Cell growth rates were evaluated by counting cell numbers. TGF-β1 was added on day 1. *C*, Chamber migration assay. TGF-β1 stimulation for 12 h. *D*, Wound healing assay. TGF-β1 stimulation for 24 h. Quantification is shown on the right. *Scale bars*: 100 µm (*C*) and 200 µm (*D*). Error bars represent SD (n=5, for *C, D*). The P values were determined by Student's *t*-test. *, $p < 0.01$. One representative result from two independent experiments is shown (*B, C, D*).

Table S1. Primers used for quantitative real-time PCR

	Forward (5'→3')	Reverse (5'→3')
ARHGAP24	TTGTGGCTGTGCTGTTTGTG	GCCTCGCAAAAGCAAAACTG
GAPDH	GAAGGTGAAGGTCGGAGTC	GAAGATGGTGATGGGATTTC
SERPINE1	GGCTGACTTCACGAGTCTTTCA	ATGCGGGCTGAGACTATGACA
